# Supplementary material for: Profiling of Tryptophan Metabolic Pathways in the Rat Fetoplacental Unit during Gestation
Source: Int J Mol Sci. 2020 Oct 14;21(20):7578. doi: 10.3390/ijms21207578 (PMC7589826; doi:10.3390/ijms21207578)
Supplement: Supplementary file 1 [file ijms-21-07578-s001.pdf]

Profiling of tryptophan metabolic pathways in the rat fetoplacental unit during gestation

SUPPLEMENTARY INFORMATION

Table 1. List of Predesigned Probe Assays (FAM and HEX) Used in qPCR and ddPCR Analysis. All Primers Were Obtained from Thermo Fisher Scientific, Waltham, MA, USA, except the HEX Assay, Which Was Obtained from BioRad, Hercules, CA, USA.

Gene symbol	Gene name	Assay ID
Tph1	tryptophan hydroxylase 1	Rn01476867_m1
Tph2	tryptophan hydroxylase 2	Rn00598017_m1
Mao-a	monoamine oxidase A	Rn01430950_m1
Mao-b	monoamine oxidase B	Rn00566203_m1
Pts	6-pyruvoyltetrahydropterin synthase	Rn00568123_m1
Spr	sepiapterin reductase	Rn01425678_g1
Ddc	dopa decarboxylase	Rn01401189_m1
Aanat	aralkylamine N-acetyltransferase	Rn00664873_g1
Asmt	acetylserotonin O-methyltransferase	Rn00595341_m1
Ido1	indoleamine 2,3-dioxygenase 1	Rn01482210_m1
Ido2	indoleamine 2,3-dioxygenase 2	Rn01482543_m1
Tdo2	tryptophan 2,3-dioxygenase	Rn00574499_m1
Kmo	kynurenine 3-monooxygenase	Rn01411937_m1
Kyat1	kynurenine aminotransferase 1	Rn01439192_m1
Kynu	Kynureninase	Rn01449532_m1
Haao	3-hydroxyanthranilate 3,4-dioxygenase	Rn01469327_m1
Qprt	quinolinate phosphoribosyltransferase	Rn01506918_g1
Slc3a2	solute carrier family 3 member 2	Rn01759899_g1
Slc6a4	solute carrier family 6 member 4	Rn00564737_m1
Slc7a5	solute carrier family member 5	Rn00569313_m1
Slc7a8	solute carrier family 7 member 8	Rn00584909_m1
Slc22a3	solute carrier family 22 member 3	Rn00570264_m1
	**Reference genes**	
Ywhaz	tyrosine 3-monooxygenase/tryptophan 5-monooxygenase activation protein, zeta	Rn00755072_m1<br>qRnoCIP0050810 (HEX)
Gapdh	glyceraldehyde-3-phosphate dehydrogenase	Rn01775763_g1

Table 2. List of Antibodies Used for Western Blot and Immunohistochemistry Analysis.

Target protein	Product name	Host	Distributor	Cat. No.	Dil.	Use
**SLC6A4**	Anti-serotonin transporter antibody	Rabbit	Abcam, Cambridge, UK	Ab181034	1:500	WB
**SLC6A4**	Anti-serotonin transporter antibody	Rabbit	Thermo Fisher Scientific, Waltham, MA, USA	PA5-50624	1:50	IHC
**SL22A3**	Anti-SLC22A3/OCT3	Rabbit	Bioworld Technology, Inc., Bloomington, MN, USA	BS3359	1:500	WB
**MAO-A**	Anti-monoamine oxidase	Rabbit	Abcam, Cambridge, UK	Ab126751	1:500	WB
					1:50	IHC
**IDO**	Anti-IDO antibody	Rabbit	LS-Bio, Seattle, WA, USA	LS-B13596	1:500	WB
**IDO**	Anti-IDO antibody	Rabbit	Thermo Fisher Scientific, Waltham, MA, USA	PA5-79437	1:50	IHC
**TPH**	Anti-TPH antibody	Rabbit	Thermo Fisher Scientific, Waltham, MA, USA	PAI-777	1:100	WB
					1:50	IHC
**Secondary antibody**	Anti-rabbit Immunoglobulins/HRP	Swine	Dako, Carpinteria, CA, USA	P0217	1:20000	WB
**Reference protein**						
**β-actin**	Anti-beta actin antibody	Mouse	Abcam, Cambridge, UK	Ab8226	1:10000	WB
**Secondary antibody**	Anti-mouse Immunoglobulins/HRP	Rabbit	Dako, Carpinteria, CA, USA	P0260	1:20000	WB
